# Comparison between anterolateral thigh free flap and jejunal flap for tissue reconstruction in patients underwent resection of pharyngoesophageal squamous cell carcinoma after radiotherapy failure: a retrospective study

**DOI:** 10.1186/s12893-021-01349-2

**Published:** 2021-11-02

**Authors:** Si-Lu Sun, Bing Zhong, Sui-zi Zhou, Jun Liu, Ya-Feng Liu, Shi-Xi Liu, Fei Chen

**Affiliations:** 1 State Key Laboratory of Oral Diseases, National Clinical Research Center for Oral Diseases, Chinese Academy of Medical Sciences Research Unit of Oral Carcinogenesis and Management, West China Hospital of Stomatology, Sichuan University, Chengdu, 610041 Sichuan People’s Republic of China; 2 Department of Otolaryngology Head and Neck Surgery, West China Hospital of Sichuan University, 37Guoxue Lane, Chengdu, 610041 Sichuan Province People’s Republic of China; 3 Department of Otolaryngology, Zhujiang Hospital, Southern Medical University, Guangzhou, 510000 Guangdong People’s Republic of China

**Keywords:** Pharyngoesophageal squamous cell carcinoma, Anterolateral thigh free flap, Jejunal flap, Radiotherapy failure, Reconstruction

## Abstract

**Background:**

Anterolateral thigh (ALT) free flap and jejunal flap (JF) were commonly used in tissue reconstruction for pharyngoesophageal squamous cell carcinoma (PESCC) with worsening tissue adhesion and necrosis after radiotherapy failure. However, the results of tissue reconstruction and postoperative complications of these two flaps are controversial. The purpose of this study was to compare outcomes between group ALT free flap and group JF in PESCC after radiotherapy failure.

**Methods:**

Intraoperative information and postoperative outcomes of patients with PESCC after radiotherapy failure who underwent ALT and JF reconstruction from January 2005 to December 2019 were compared and analyzed.

**Results:**

The defect size of ALT (Numbers, 34) and JF (Numbers, 31) was 36.19 ± 11.35 cm^2^ and 35.58 ± 14.32 cm^2^ (p = 0.884), respectively. ALT and JF showed no significant difference in operation time (p = 0.683) and blood loss (p = 0.198). For postoperative outcomes within 30 days both in recipient site and donor site including wound bleeding, wound dehiscence, wound infection, and pharyngocutaneous fistula, ALT free flap and JF showed similar results. Flap compromise (Numbers, 2 VS.3, p = 0.663), flap take backs (Numbers, 1 VS.1, p = 1.000), partial flap failures (Numbers, 4 VS.2, p = 0.674), and total flap failures (Numbers, 0 VS.0, p = 1.000) showed no difference between the two groups. In addition, no significance was found in hypoproteinemia between the two groups (Numbers, 4 VS.2, p = 0.674). ALT free flap was not statistically different from JF in the incidence of dysphagia at the postoperative 6 months (Numbers of liquid diet, 5VS.5; Numbers of partial tube feeding, 6VS.7; Numbers of total tube feeding, 3VS.1, p = 0.790) and 12 months (Numbers of liquid diet, 8VS.7; Numbers of partial tube feeding, 8VS.7; Numbers of total tube feeding, 5VS.5, p = 0.998). The cause of dysphagia not found to differ between the two groups both in postoperative 6 months (p = 0.814) and 12 months (p = 0.845).

**Conclusion:**

Compared with JF, ALT free flap for PESCC patients after radiotherapy failure showed similar results in postoperative outcomes. ALT free flap may serve as a safe and feasible alternative for PESCC patients after radiotherapy failure.

## Introduction

PESCC is a malignant tumor of the head and neck with poor prognosis that invades the hypopharynx [1]. Large tissue defects caused by tumor resection are always a challenge for surgeons [2, 3]. For the patients with radiotherapy failure, the more severe the tissue adhesion, the greater the possibility of lymph node metastasis and distant metastasis [4]. Therefore, extensive invasion makes it impossible to effectively maintain swallowing function after the resection of PESCC lesions, and tracheotomy and neck tissue reconstruction are required to save the lives of the patients [5, 6].

JF and gastric pull-up have been regarded as routine surgical and repair methods for PESCC for a long time [7, 8]. However, the scar contracture caused by radiotherapy on the muscles, blood vessels and nerves within the head and neck tissue limits the use of the methods. At the same time, ALT free flap has become increasingly prominent for the repair and reconstruction of head and neck tumors in recent years [9]. Its moldability has become a huge advantage in rebuilding tissue defects [10, 11]. The present study aimed to analyze the outcomes of short-term and long-term postoperative complications following ALT or JF reconstruction for patients with PESCC after radiotherapy failure.

## Patients and methods

This study was approved by the ethics committee of West China Hospital. All patients signed informed consent. The study protocol is performed in accordance with the relevant guidelinesIn this study, the patients with PESCC after radiotherapy failure who underwent tumor resection at West China Hospital, Chengdu between January 2005 to December 2019 were enrolled. All the patients underwent ALT or JF reconstruction. The patients underwent preoperative blood examination, laryngoscopy biopsy, head and neck computed tomography (CT) or magnetic resonance imaging (MRI), chest X-ray or CT, and abdominal ultrasound. History of radiotherapy failure was determined by oral questioning, physical examination, head and neck imaging examination, and treatment history. The diagnosis was confirmed by postoperative pathology. Patients who met the following inclusion criteria were selected: male or female, aged 18–75 years; pathology-confirmed PESCC; failure of radiotherapy; no distant metastasis; and no peripheral vascular diseases of the lower limbs, such as arterial stenosis, arterial embolism, thromboangiitis obliterans, and deep venous thrombosis. The exclusion criteria were severe dysfunction of the heart, kidneys or other organs and a history of other malignancies.

### Surgical techniques

**Fig. 1:**
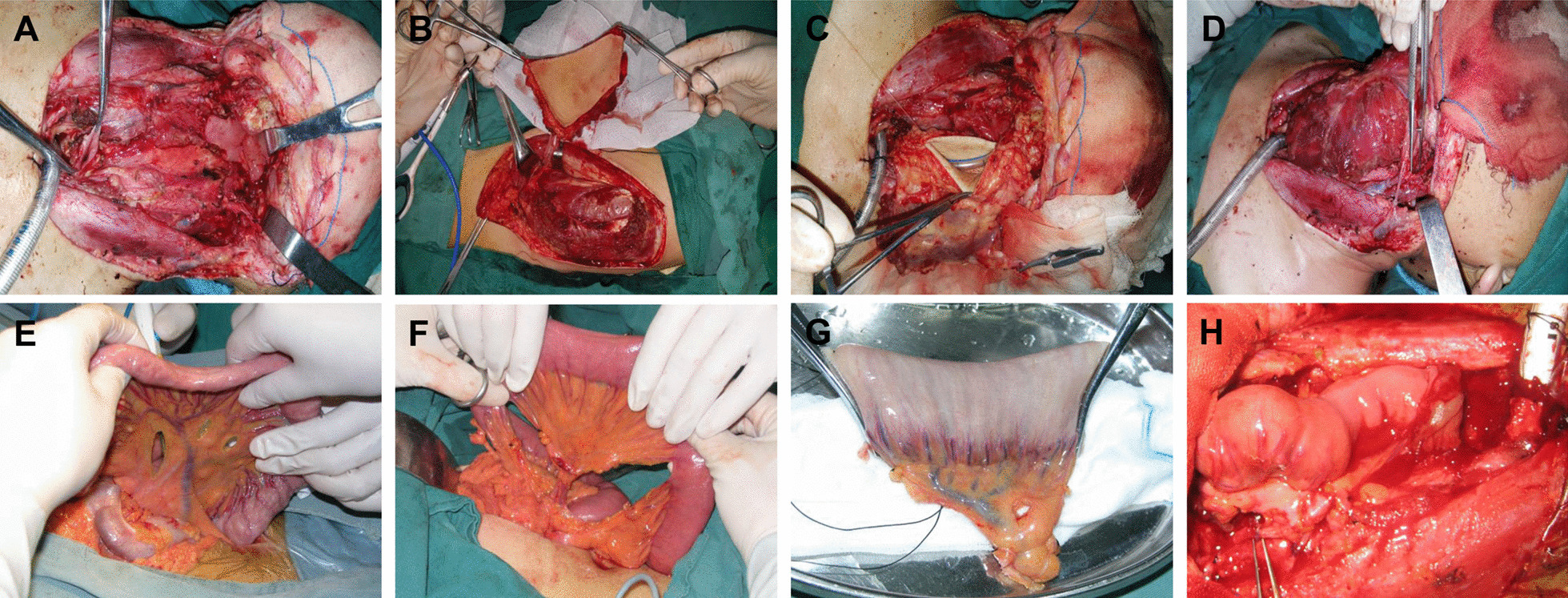
Surgical procedure of ALT (**A**–**D**) and jejunal flap (E–H). **A** Resection of tumor. **B** Cut ALT. **C** Reconstruction defect. **D** Anastomotic vessel. **E**–**G** Cut jejunal flap. **H** Reconstruction of defects and anastomoses

All patients were evaluated by the surgeons for gastrointestinal condition and lower limb health, and appropriate repair options were selected. The patient was placed in the supine position after general intravenous anesthesia. AU-shaped incision was made at the level of the neck to the prevertebral fascia to expose and remove the tumor. The lymph nodes were also dissected (Fig. 1A). The ALT flap (The fascia latissima and myofascial fascia were obtuse separated after dissecting the skin and deep fascia, followed by dissection of the musculocutaneous vascular bundle and dissection of the flap) (Fig. 1B, C) or the JF(Pedicled jejunum flap: we made a longitudinal incision in the abdomen to remove the jejunum and blood vessels of appropriate length and anastomosed the remaining jejunum stump) (Fig. 1E–G) was removed to match the size of the defect. We made the ALT flap into a tube with the skin as the inner wall. The ALT or JF was stitched between the remaining pharynx and esophagus. Arterio-venous anastomoses was performed under a microscope (Artery:superior thyroid artery, arteria lingualis, facial artery, fransverse cervical artery; Vein: facial vein, posterior facial vein, vena jugularis externa, vena jugularis interna), and the degree of vascular patency was assessed by the blood vessel color and the skin temperature (Fig. 1D and H). Finally, a drainage tube was placed on the neck for draining blood and secretions. All patients underwent total laryngectomy and permanent tracheostomy because of laryngeal involvement. The skin temperature of the flap area was measured twice daily after surgery and the temperature difference between the operated area and the unoperated area was determined to measure the survival of the flap. In addition, ultrasound was performed in patients with suspected flap necrosis to confirm vascular status.

### Assessment

Blood tests were performed in 1, 3, 5, and 7 days after surgery. Follow-up was performed in every 3 months in the first year after discharge and every 6 months thereafter. Blood tests, laryngoscopy, head and neck CT or MRI were performed on each patient at each follow-up visit. A barium swallow test was usually performed 2 or 4 weeks after the transplant. A liquid diet was initiated if no leakage was identified in the barium swallow test. If the fluid diet went smoothly, the gastric tube was removed, and a solid diet was initiated; otherwise, the gastric tube was retained. Dysphagia is defined as difficulty swallowing solid food and receiving only liquids or relying partly or entirely on tube feeding. Radiotherapy failure was defined as follows: no reduction of tumor after radiotherapy; or tumor progression includes enlargement of tumor size, lymph node metastasis or distant metastasis after radiotherapy; or recurrence after completed radiotherapy.

### Statistical analysis

SPSS 22.0 statistical software was used to analyze the data and the experimental results. All the data were expressed as means ± standard deviation (SD).The data with anormal distribution were analyzed by student's t-test, and the signed-rank test was used to analyze the data with nonnormal distribution. P < 0.05 indicates statistical significance.

## Results

In total, 65 patients with PESCC at the age of 62.31 ± 12.57 and 59.48 ± 12.56 years who underwent ALT flap surgery (n = 34; 25 males and 9 females) or JF surgery (n = 31; 23 males and 8 females) were enrolled in this study. 22 and 20 patients had a history of smoking, respectively. 20 and 22 had a history of alcohol abuse, respectively. All patients had pathological T stage IV with lymph node metastasis, but no distant metastasis. The low, moderate and poor differentiation of the ALT and JF were 0 vs. 0, 28 vs. 26 and 6 vs. 5, respectively. 4 and 6 patients had a history of throat surgery in ALT and JF, respectively. Additionally, the number of the patients with diabetes and hypertension was 8 and 9 in the ALT group while 6 and 5 in the JF group, respectively (Table 1).

**Table 1:** Patient characteristics

Variables	Number /Mean ± SD		p
	ALT(34)	JF(31)	
Age, y	62.31 ± 12.57	59.48 ± 12.56	0.755
Sex			1.000
Male	25	23	
Female	9	8	
Smoking	22	20	1.000
Alcohol	20	22	0.436
Pathological T staging (I/II/III/IV)	0/0/0/34	0/0/31	1.000
Pathological N staging (yes/no)	34/0	31/0	1.000
Pathological M staging (yes/no)	0/34	0/31	1.000
Differentiation (well/moderate/poor)	0/28/6	0/26/5	1.000
History of throat surgery	4	6	0.500
Chemotherapy	5	6	0.745
Diabetes	8	6	0.768
Hypertension	9	5	0.382

As shown in Table 2, the operation times for the ALT flap and JF reconstructions were 365.44 ± 81.57 and 399.58 ± 86.67 min (p = 0.683) with blood losses of 699.37 ± 118.61 and 754.43 ± 104.32 ml (p = 0.198), respectively. The defect size in the two groups was 36.19 ± 11.35 cm^2^ and 35.58 ± 14.32 cm^2^, respectively (p = 0.884). Ischemia time of free flap was 86.55 ± 18.43 min and 91.66 ± 21.31 min, respectively (p = 0.692). The size of the repair materials for the ALT flap reconstruction was 126.62 ± 31.27 cm^2^ and the length of JF was 12.84 ± 4.91 cm. Futhermore, the number of the patients who presented postoperative complications in recipient site and donor site with wound bleeding, wound dehiscence, and wound infection in the ALT flap and JF reconstructions were 5 vs. 4 (p = 1.000), 2 vs. 3 (p = 0.647), 7 vs. 8 (p = 0.770), and 1 vs. 1 (p = 1.000), 0 vs. 0 (p = 1.000), 1 vs. 0 (p = 1.000), respectively. The pharyngocutaneous fistula (p = 0.527), flap compromise (p = 0.663), flap take backs (p = 1.000), partial flap failures (p = 0.674) and total flap failures (p = 1.000) in recipient site were 2 vs. 3, 1 vs. 1, 4 vs. 2, 0 vs. 0, respectively. Number of hypoproteinemia in ALT flap and JF were 23 and 21, respectively (p = 0.408). In addition, the Hospital length of stay (LOS) in two groups were 18.52 ± 6.17 vs. 20.39 ± 7.58 (p = 0.718). ICU stay showed no significant difference between the two groups (Numbers/Days, 1/3, 1/5, p = 1.000). Six months after reconstructions, the number of patients with dysphagia in group ALT flap and group JF was 5 vs. 5 (liquid diet), 6 vs. 7 (partial tube feeding), 3 vs. 1 (total tube feeding), respectively (p = 0.790). The number of patients with dysphagia in two groups was 8 vs. 7 (liquid diet), 8 vs. 7 (partial tube feeding), and 5 vs. 5(total tube feeding) at the 12th month (p = 0.962), respectively. The causes of dysphagia in group ALT flap and group JF with scar contracture, anastomotic stricture, tumor recurrence in 6 months were 3 vs. 4, 3 vs. 3, and 1 vs. 0, respectively (p = 0.814). The above indicators in two groups were 6 vs. 5, 3 vs. 4, and 4 vs. 3 at the 12 months (p = 0.845). Additionally, the remaining patients had satisfactory swallowing function. None of the patients received JF reconstruction had abdominal complications, including ileus, hernia, bowel injury, and abdominal bleeding.

**Table 2:** Intraoperative data and postoperative outcomes

Variables	Number/Mean ± SD		p
	ALT(34)	JF(31)	
Intraoperative data			
Operation time (min)	365.44 ± 81.57	399.58 ± 86.67	0.683
Blood loss (ml)	699.37 ± 118.61	754.43 ± 104.32	0.198
Defect size (area, cm^2^)	36.19 ± 11.35	35.58 ± 14.32	0.884
Flap size (area, cm^2^/length, cm)	131.55 ± 32.45	14.67 ± 4.43	
Ischemia time of free flap(min)	66.55 ± 18.43	61.66 ± 21.31	0.692
Postoperative outcomes within 30 days Recipient site			
Wound bleeding	5	4	1.000
Wound dehiscence	2	3	0.647
Wound infection	7	8	0.770
Pharyngocutaneous fistula	5	7	0.527
Flap compromise	2	3	0.663
Flap take backs	1	1	1.000
Partial flap failures	4	2	0.674
Total flap failures	0	0	1.000
Donor site			
Wound bleeding	1	1	1.000
Wound dehiscence	0	0	1.000
Wound infection	1	0	1.000
Hypoproteinemia	23	21	0.408
Hospital LOS	18.52 ± 6.17	20.39 ± 7.58	0.718
ICU stay(Numbers)	1	1	1.000
Days	3	5	
Dysphagia at postoperative 6 months			0.790
Liquid diet	5	5	
Partial tube feeding	6	7	
Total tube feeding	3	1	
Cause			0.814
Scar contracture	5	4	
Anastomotic stricture	3	3	
Tumor recurrence	1	0	
Dysphagia at postoperative 12 months			0.998
Liquid diet	8	7	
Partial tube feeding	8	7	
Total tube feeding	5	5	
Cause			0.845
Scar contracture	6	5	
Anastomotic stricture	3	4	
Tumor recurrence	4	3	

## Discussion

Radiotherapy has become an important modality to treat PESCC. However, for some advanced patients, failure of radiotherapy will cause serious tissue adhesion, and increase the difficulty for radical surgery [12]. Moreover, pharyngoesophageal squamous cell carcinoma surgical resection causes large tissue defects, and necrosis and contracture of tissue caused by radiotherapy increases the difficulty of defect reconstruction. The extent of surgical removal of the tumor makes vascular, nerve repair and tissue reconstruction extremely complex. Restoring the physiological functions of the patients' throat, such as swallowing, is a huge challenge [13, 14]. With the development of microsurgery, tissue transfer, and repair methods, including the use of ALT and JF have replaced gastric pull-up in reconstruction [15–17].

The jejunal valve has similar lubrication and peristaltic functions to the hypopharynx and esophagus, which is suitable to repair the tissue defect caused by the treatment of PESCC [18]. While the ALT flap, with advantages of soft tissue, rich blood supply, and wide resectable range, has also provided the reconstructive surgeons with an alternative treatment option for complex head and neck defects. However, in our 12-year retrospective study, early surgical repair method was primarily based on JF, whereas in the last five years, ALT had gradually become the primary repair material, probably because the size of the ALT flap simplifies the operation and allows for the adjustment of the size of surface defects on the cutting surface, especially in the head and neck tumors.

It is well known that differences in the time of surgery and the amount of blood loss are also important factors in determining the type of surgery which may affect albumin level in blood. However, for PESCC patients after radiotherapy failure, tissue adhesion makes surgery more complicated, leading to prolonged operation time and increased bleeding, and the time saving of the removal of repair materials and tissue reconstruction becomes crucial. Our results suggested that although ALT and JF were harvested at different sites, there was no difference in operative time or blood loss, while the occurrence of hypoproteinemia was also similar in both ways.

For PESCC, the severity of postoperative complications makes it important to reduce the complication rate for the success of surgery. Postoperative wound infection and bleeding are critical to the survival of free skin flap and microvascular circulation, which determine the short-term prognosis of patients. Our results suggested that there were no differences in wound infection, bleeding and pharyngeal fistula between ALT and JT, which also indicated that short-term complications were not the decisive factors in determining the preference of two repair methods. Although many studies have confirmed that the JF can be used for the reconstruction of head and neck defects, abdominal complications such as intestinal obstruction and bleeding are still the main issue of this flap. The study of Yu et al. demonstrated that one patient developed abdominal hemorrhage, and one patient developed intestinal obstruction due to intussusception in 202 patients with hypopharyngeal defects after jejunal repair [19]. However, the patients in our study who underwent JF repair did not experience abdominal complications.

The primary goal of tissue reconstruction after tumor resection is to repair the continuity of the pharyngeal ducts to maximize the recovery of breathing and swallowing functions. Previous studies have shown that JF is satisfactory in repairing the swallowing function of head and neck tumors [15, 17, 20, 21]. Tan et al. found that a single ALT fascia flap could well repair complex hypopharyngeal, esophageal and anterior cervical skin defects in patients with head and neck tumors [22]. ALT has also achieved excellent results in the reconstruction of head and neck tissues in recent years [18]. In view of the possibility that mass tissue resection caused by tissue adhesion and necrosis of PESCC after radiotherapy failure may have a greater impact on postoperative swallowing function, we evaluated ALT and JF on swallowing function at the 6th and the 12th month postoperatively to improve the accuracy of analyzing the effects of ALT and JF on postoperative swallowing function reconstruction. Our results suggested that although the number of patients with dysphagia including liquid diet, partial tube feeding and total tube feeding in ALT and JF increased gradually with the protract of time, there was no statistical significance between the two groups, which was similar to the results of previous study [23]. However, in previous study, the results of Chan et al. suggested that JF reconstruction of peripharyngectomy defects can achieve better functional outcomes than ALT, which may be different from our results, possibly because our study included patients after radiotherapy and had a small sample size [19]. It is worth mentioning that the cause of postoperative dysphagia in the JF group may be stenosis caused by hypoperfusion at the stump anastomosis, and vasopression may provide better results, which may be helpful for our future treatment.

However, this study has the following limitations: firstly, the sample size may limit the credibility of the conclusion. Secondly, the number of times each patient received radiation therapy was different, which also had an impact on the outcome. In the future, we will expand our sample size and further confirm the results in experiments.

In conclusion, we investigated ALT and JF reconstruction for tissue defect in patients with PESCC after radiotherapy failure. The results showed that the reconstruction with ALT flap was comparable with JF in postoperative complications. ALT flap and JF may all serve as equally safe and feasible alternatives for the patients with PESS after radiotherapy failure.

## Data Availability

All data generated or analysed during this study are included in this published article.

## Abbreviations

ALT: Anterolateral thigh
JF: Jejunal flap
PESCC: Pharyngoesophageal squamous cell carcinoma
CT: Computed tomography
MRI: Magnetic resonance imaging
SD: Standard deviation
